# Implementation of sepsis bundles in public hospitals in Brazil: a prospective study with heterogeneous results

**DOI:** 10.1186/s13054-017-1858-z

**Published:** 2017-10-31

**Authors:** Flavia Ribeiro Machado, Elaine Maria Ferreira, Pierre Schippers, Ilusca Cardoso de Paula, Letícia Sandre Vendrame Saes, Francisco Ivanildo de Oliveira, Paula Tuma, Wilson Nogueira Filho, Felipe Piza, Sandra Guare, Cláudia Mangini, Gustavo Ziggiatti Guth, Luciano Cesar Pontes Azevedo, Flavio Geraldo Resende Freitas, Jose Luiz Gomes do Amaral, Nacime Salomão Mansur, Reinaldo Salomão, on behalf of the SPDM against sepsis project

**Affiliations:** 1 Anesthesiology, Pain and Intensive Care Department, Federal University of São Paulo, São Paulo, SP Brazil; 2 Latin American Sepsis Institute, São Paulo, SP Brazil; 3 Latin American Sepsis Institute, Universidade Federal de São Paulo, Rua Napoleão de Barros, 715 - 6° andar, Vila Clementino, 04024-002 São Paulo, SP Brazil; 4 Sociedade Paulista para o Desenvolvimento da Medicina (SPDM), São Paulo, SP Brazil; 5 Infectious Disease Department, Federal University of São Paulo, São Paulo, SP Brazil

**Keywords:** Sepsis, Bundles, Septic shock, Developing countries

## Abstract

**Background:**

Public hospitals in emerging countries pose a challenge to quality improvement initiatives in sepsis. Our objective was to evaluate the results of a quality improvement initiative in sepsis in a network of public institutions and to assess potential differences between institutions that did or did not achieve a reduction in mortality.

**Methods:**

We conducted a prospective study of patients with sepsis or septic shock. We collected baseline data on compliance with the Surviving Sepsis Campaign 6-h bundles and mortality. Afterward, we initiated a multifaceted quality improvement initiative for patients with sepsis or septic shock in all hospital sectors. The primary outcome was hospital mortality over time. The secondary outcomes were the time to sepsis diagnosis and compliance with the entire 6-h bundles throughout the intervention. We defined successful institutions as those where the mortality rates decreased significantly over time, using a logistic regression model. We analyzed differences over time in the secondary outcomes by comparing the successful institutions with the nonsuccessful ones. We assessed the predictors of in-hospital mortality using logistic regression models. All tests were two-sided, and a *p* value less than 0.05 indicated statistical significance.

**Results:**

We included 3435 patients from the emergency departments (50.7%), wards (34.1%), and intensive care units (15.2%) of 9 institutions. Throughout the intervention, there was an overall reduction in the risk of death, in the proportion of septic shock, and the time to sepsis diagnosis, as well as an improvement in compliance with the 6-h bundle. The time to sepsis diagnosis, but not the compliance with bundles, was associated with a reduction in the risk of death. However, there was a significant reduction in mortality in only two institutions. The reduction in the time to sepsis diagnosis was greater in the successful institutions. By contrast, the nonsuccessful sites had a greater increase in compliance with the 6-h bundle.

**Conclusions:**

Quality improvement initiatives reduced sepsis mortality in public Brazilian institutions, although not in all of them. Early recognition seems to be a more relevant factor than compliance with the 6-h bundle.

**Electronic supplementary material:**

The online version of this article (doi:10.1186/s13054-017-1858-z) contains supplementary material, which is available to authorized users.

## Background

Although sepsis is still a leading cause of mortality worldwide, mortality rates are decreasing [1], especially in developed countries [2, 3]. However, low- and middle-income countries (LMICs) are responsible for a significant portion of the sepsis burden [4], and mortality rates are still very high in these settings [5–8]. Quality improvement initiatives are successful in reducing fatality rates in high-income countries [9], but there are only a few reports of such initiatives in LMICs [10–15]. In Brazil, some studies have already shown higher mortality rates in public institutions than in private ones [16, 17]; also, public institutions have worse compliance with treatment quality indicators, and mortality reductions in quality improvement initiatives seem not to be sustained [16, 18].

Public hospitals in emerging countries pose a challenge to quality improvement initiatives. These facilities usually have considerable limitations, including infrastructure issues; low availability of resources [19]; low availability of intensive care unit (ICU) beds [20]; a shortage of healthcare professionals [21]; inadequate staff qualification and high turnover [22, 23]; and overcrowding, especially in the emergency department (ED). One of the main barriers is the low awareness of sepsis [23], leading to late recognition with a consequent delay in intervention and increased lethality [16, 24]. Therefore, one of the main goals of any quality improvement initiative should be to train hospital staff to identify those patients in earlier phases. Early recognition and training are associated with a reduction in the severity of illness among patients with sepsis, as well as with lower severity scores and less organ dysfunction at the time of the sepsis diagnosis [15].

Therefore, we aimed to assess whether a quality improvement initiative in a network of public hospitals in Brazil would decrease mortality and the factors that would be associated with this reduction. On the basis of our previous experience in public hospitals [18], we anticipated that the success of the initiative would be heterogeneous among the hospitals; therefore, our secondary objective was to identify the quality indicators associated with a reduction in mortality rates.

## Methods

We conducted a prospective study in a network of nine public hospitals with a central administration. The institutions were instructed to include clinical and sepsis management variables from all patients admitted to the ED, wards, or ICUs with a diagnosis of sepsis or septic shock in a centralized electronic database [25]. We defined sepsis as the presence of infection complicated by acute organ dysfunction (previously called *severe sepsis*) [17], as detailed in the Additional file. Septic shock was defined by the presence of refractory hypotension with the need for vasopressors. Patients could be included in the database only once in the same hospital admission. Patients receiving end-of-life care were excluded. The research and ethics committee of Universidade Federal de São Paulo approved the study (1387/10) and waived the need for informed consent because of the nature of the study.

### Intervention

The quality improvement initiative was conducted as suggested by the Latin American Sepsis Institute (LASI) [15]. It was divided into two phases. All institutions were asked to select a case manager responsible for the project. After training, each case manager was instructed to collect data on all cases of sepsis and septic shock based on an active search for those patients in the main hospital areas over 3 months as a baseline. The LASI suggested strategies for the active search, such as performing an audit of all prescribed antibiotics. Data on the patients’ characteristics, compliance with the Surviving Sepsis Campaign (SSC) 6-h bundle (Additional file 1: Table S1), and hospital mortality were collected. To assess compliance, we considered the moment of sepsis recognition by the healthcare team, instead of the time of organ dysfunction onset, as time zero. During this baseline period, all institutions were also asked to create a sepsis team. The institutions had to develop treatment protocols, a guide for empiric antibiotic therapy, and a screening tool based on the presence of signs of systemic inflammatory response syndrome or organ dysfunction (reduced level of consciousness, dyspnea or oxygen requirement, hypotension, or low urine output) that could be used in the ED, wards, and ICUs. They were also asked to establish a routine for laboratory tests, allowing for more rapid reporting of results, as well as a routine for timely supply of antibiotics to all hospital sectors.

In the second phase, we started a continuous education program that aimed to train all nurses and physicians. All hospital staff were invited to participate in an 8-h course provided by the LASI with basic knowledge about sepsis recognition and treatment. Video lectures and an e-learning course were available at the LASI website. Educational materials, such as flowcharts, panels, and folders, were widely distributed in all institutions. Data collection was continued throughout the 2-year intervention, and each institution received a quarterly performance report from the LASI. The report summarized compliance with the bundle items, as well as hospital mortality data for each hospital sector, and provided benchmarking with the network and the entire LASI database. The results were discussed with the sepsis team and other healthcare professionals in quarterly joint meetings with the LASI team; at these meetings, opportunities for improvement and new strategies for optimization were discussed. Although LASI provided all tools and information to allow a similar intervention in all sites, ultimately local implementation was highly dependent on each institution.

### Data collection

All information was collected using dedicated software that was specifically developed for this project, and there were automatic checks of data entry to improve the data completeness and consistency. All data were input prospectively by the case manager of the institution. The entire database was confidential, and each institution had access to only its own data.

We collected data on the patients’ demographics; sepsis characteristics; location at organ dysfunction onset; time to sepsis diagnosis; and severity of illness as assessed by the Acute Physiology and Chronic Health Evaluation II (APACHE II) score, Sequential Organ Failure Assessment (SOFA) score, and total number of organs with dysfunction at the time of the sepsis diagnosis. We also collected data on the compliance with the SSC 6-h bundle. We registered ICU admission in only the first 24 h of the sepsis diagnosis. Patients were followed until hospital discharge.

### Outcomes

The primary outcome was hospital mortality over time. The predefined secondary outcomes were the time to sepsis diagnosis and compliance with the entire 6-h bundles throughout the intervention. As a secondary outcome, we also considered compliance with the 6-h bundle items that are required for all patients, including lactate sampling, blood cultures, and antibiotic administration. On the basis of our previous study [15], we hypothesized that the implementation would improve sepsis awareness with a reduction in the time to sepsis diagnosis and disease severity. Therefore, we included the percentage of sepsis in all patients, APACHE II score, SOFA score, and total number of organs with dysfunction as secondary outcomes.

### Definitions

On the basis of our previous experience with the quality improvement program in the LASI [18], we expected that the mortality rates would not decrease in some institutions. Thus, we defined successful institutions as those where the mortality rates decreased significantly over time, and those in which the mortality rates did not drop were defined as nonsuccessful institutions. We used logistic regression with quarter and institution as explanatory variables and death as a dependent variable. The institutions that presented a statistically significant reduction in mortality OR according to quarter change were considered as the successful institutions.

### Statistical analysis

The categorical variables are expressed as absolute numbers and percentages, and the continuous variables are reported as measures of central tendency and dispersion according to their distribution (mean [SD] or median [IQR]) as assessed by the Kolmogorov-Smirnov test.

#### Primary and secondary outcomes

We analyzed the primary and secondary outcomes throughout the intervention using generalized linear models to assess their trend over quarter changes in the entire population [26]. Generalized linear models were used, considering both the quarter and institutions as explanatory variables, accounting for the cluster effect. We also aimed to analyze differences over time in both the primary and secondary outcomes by comparing the successful institutions with the nonsuccessful ones. We also calculated the rate of decline (quarter mean time to sepsis diagnosis/quarter mean time to sepsis diagnosis + 1 in the next quarter), using generalized linear models with gamma distribution and log-link function to evaluate if the rate of decline was different between the successful and nonsuccessful institutions.

#### Prognostic factors associated with mortality

In the univariate analysis to assess the prognostic factors for mortality, we used Fisher’s exact test or the chi-square test for categorical data and the Mann-Whitney *U* test for continuous data without a normal distribution. We assessed the predictors of in-hospital mortality using logistic regression models with random effects at the institution level to account for the clustering effect [26]. We included in the model all variables with a *p* value < 0.05 in the univariate analysis and institution type (successful and nonsuccessful); however, we did not include lactate ≥ 4 mmol/L, owing to excessive missing data and the individual dysfunctions because they were already assessed in the SOFA score. Because we considered compliance with fluids and vasopressors a variable with special interest, we ran a second model including this variable. We also ran a sensitivity analysis excluding the institutions that did not include patients in the last quarters of the intervention. We present the general OR with 95% CI for those variables and, when there was a significant difference between institutions, individually according to the institution type. All tests were two-sided, and a *p* value < 0.05 indicated statistical significance. Analyses were performed using R 3.2.2 software (2014; R Core Team, Vienna, Austria).

## Results

From September 2010 to August 2012, we included 3435 patients from the 9 participating hospitals. The baseline characteristics of the patients throughout the intervention are available in Table 1, and the individual results are provided in Additional file 1: Table S2. There was no major change in the patients’ characteristics, except for discreet changes in the frequency of some comorbidities, the urinary tract as a source of infection, and the location at sepsis presentation.

**Table 1:** Study population characteristics throughout the intervention

Variable	Baseline (*n* = 384)	Second quarter (*n* = 288)	Third quarter (*n* = 422)	Fourth quarter (*n* = 531)	Fifth quarter (*n* = 563)	Sixth quarter (*n* = 492)	Seventh quarter (*n* = 398)	Eighth quarter (*n* = 357)	*p* Value^a^
Age, years	61.0 (46.0–75.2)	62.5 (47.0–75.0)	63.0 (49.2–75.0)	61.0 (48.0–74.0)	60.0 (46.0–74.0)	60.0 (44.0–74.0)	61.0 (46.2–74.0)	62.0 (50.0–74.5)	0.133
Male sex	223 (58.1)	157 (54.5)	240 (56.9)	293 (55.2)	326 (57.9)	270 (54.9)	227 (57.0)	197 (55.2)	0.802
Comorbidities									
Diabetes	98 (25.5)	71 (24.7)	108 (25.6)	141 (26.6)	148 (26.3)	111 (22.6)	90 (22.6)	75 (21.0)	0.216
Immunosuppression	66 (17.2)	38 (13.2)	81 (19.2)	96 (18.1)	95 (16.9)	89 (18.1)	88 (22.1)	85 (23.8)	0.380
COPD	41 (10.7)	28 (9.7)	41 (9.7)	35 (6.6)	44 (7.8)	24 (4.9)	20 (5.0)	23 (6.4)	0.133
CRF	43 (11.2)	37 (12.8)	55 (13.0)	81 (15.3)	64 (11.4)	43 (8.7)	42 (10.6)	42 (11.8)	<0.0001
Cancer	45 (11.7)	28 (9.7)	51 (12.1)	58 (10.9)	54 (9.6)	52 (10.6)	66 (16.8)	60 (16.8)	0.330
Arterial hypertension	183 (47.7)	134 (46.5)	186 (44.1)	234 (44.1)	243 (43.2)	185 (37.6)	143 (35.9)	142 (39.8)	0.004
Type of patient									0.343
Medical	281 (73.2)	191 (66.3)	325 (77.0)	418 (78.7)	411 (73.0)	358 (72.8)	295 (74.1)	288 (80.7)	
Surgical	103 (26.8)	97 (33.7)	97 (23.0)	113 (21.3)	152 (27.0)	134 (27.2)	103 (25.9)	69 (19.3)	
Source of infection									
Pneumonia	248 (64.6)	157 (54.5)	260 (61.6)	302 (56.9)	328 (58.3)	250 (50.8)	185 (46.5)	197 (55.2)	0.493
Urinary tract	61 (15.9)	55 (19.1)	60 (14.2)	95 (17.9)	89 (15.8)	90 (18.3)	69 (17.3)	53 (14.8)	<0.0001
Abdominal	67 (17.4)	51 (17.7)	61 (14.5)	63 (11.9)	79 (14.0)	90 (18.3)	78 (19.6)	45 (12.6)	0.893
Type of infection									0.078
Community	238 (62.0)	168 (58.3)	230 (54.5)	280 (52.7)	339 (60.2)	284 (57.7)	226 (56.8)	233 (65.3)	
Nosocomial	146 (38.0)	120 (41.7)	192 (45.5)	251 (47.3)	224 (39.8)	208 (42.3)	172 (43.2)	124 (34.7)	
Location at presentation									<0.0001
Emergency department	184 (47.9)	114 (39.6)	179 (42.4)	224 (42.2)	247 (43.9)	232 (47.2)	201 (50.5)	248 (69.5)	
Ward	145 (37.8)	129 (44.8)	184 (43.6)	221 (41.6)	219 (38.9)	174 (35.4)	141 (35.4)	65 (18.2)	
ICU	55 (14.3)	45 (15.6)	59 (14.0)	86 (16.2)	97 (17.2)	86 (17.5)	56 (14.1)	44 (12.3)	

*COPD* Chronic obstructive pulmonary disease, *CRF* Chronic renal failure, *ICU* Intensive care unit

^a^General linear models

There was a significant reduction in the mortality rates over time with *p* < 0.0001 (Table 2). This reduction was significant for patients with sepsis and septic shock. There was also an improvement in the secondary outcomes with a significant reduction in the time to sepsis diagnosis and an increased percentage of patients diagnosed with sepsis instead of septic shock. Concomitantly, there was a reduction in the disease severity, as measured by the APACHE II score, SOFA score, and total number of organs with dysfunction. The compliance with the first three required items of the 6-h bundle, as well as compliance with the entire bundle, also increased significantly.

**Table 2:** Outcomes throughout the intervention in the whole population and according to the success of the institutions

Variables	Baseline (*n* = 384)	Second quarter (*n* = 288)	Third quarter (*n* = 422)	Fourth quarter (*n* = 531)	Fifth quarter (*n* = 563)	Sixth quarter (*n* = 492)	Seventh quarter (*n* = 398)	Eighth quarter (*n* = 357)	*p* Value^a^
Hospital mortality									
All institutions	226 (58.9)	177 (61.5)	236 (55.9)	278 (52.4)	280 (49.7)	250 (50.8)	183 (46.0)	166 (46.5)	<0.0001
Successful institutions	113 (51.4)	71 (60.2)	122 (50.4)	103 (43.3)	117 (45.0)	78 (36.1)	77 (36.8)	114 (41.0)	<0.0001
Nonsuccessful institutions	113 (68.9)	106 (62.4)	114 (63.3)	175 (59.7)	163 (53.8)	172 (62.3)	106 (56.1)	52 (65.8)	
Hospital mortality: sepsis									
All institutions	85 (42.7)	76 (47.8)	108 (43.7)	155 (45.1)	125 (35.9)	118 (39.3)	97 (35.9)	85 (37.3)	0.028
Successful institutions	36 (31.9)	35 (52.2)	56 (39.2)	60 (36.1)	64 (35.2)	33 (24.8)	43 (28.5)	64 (33.3)	<0.0001
Nonsuccessful institutions	49 (57.0)	41 (44.6)	52 (50.0)	95 (53.4)	61 (36.7)	85 (50.9)	54 (45.4)	21 (58.3)	
Hospital mortality: septic shock									
All institutions	141 (76.2)	101 (78.3)	128 (73.1)	123 (65.8)	155 (72.1)	132 (68.8)	86 (67.2)	81 (62.8)	0.0020
Successful institutions	77 (72.0)	36 (70.6)	66 (66.7)	43 (59.7)	53 (67.9)	45 (54.2)	34 (58.6)	50 (58.1)	<0.0001
Nonsuccessful institutions	64 (82.1)	65 (83.3)	62 (81.6)	80 (69.6)	102 (74.5)	87 (79.8)	52 (74.3)	31 (72.1)	
Hospital mortality: ED									
All institutions	94/184 (51.1)	69/114 (60.5)	91/179 (50.8)	104/224 (46.4)	109/247 (44.1)	97/232 (41.8)	78/201 (38.8)	113/248 (45.6)	0.003
Successful institutions	63/136 (46.3)	31/55 (56.4)	50/108 (46.3)	38/110 (34.5)	53/138 (38.4)	35/116 (30.2)	34/113 (30.1)	82/207 (39.6)	<0.0001
Nonsuccessful institutions	31/48 (64.6)	38/59 (64.4)	41/71 (57.7)	66/114 (57.9)	56/109 (51.4)	62/116 (53.4)	44/88 (50.0)	31/41 (75.6)	
Hospital mortality: wards									
All institutions	99/145 (68.3)	71/129 (55.0)	116/184 (63.0)	128/221 (57.9)	116/219 (53.0)	101/174 (58.0)	67/141 (47.5)	28/65 (43.1)	0.0001
Successful institutions	29/48 (60.4)	26/42 (61.9)	54/91 (59.3)	41/77 (53.2)	33/67 (49.3)	15/47 (31.9)	22/60 (36.7)	11/35 (31.4)	<0.0001
Nonsuccessful institutions	70/97 (72.2)	45/87 (51.7)	62/93 (66.7)	87/144 (60.4)	83/152 (54.6)	86/127 (67.7)	45/81 (55.6)	17/30 (56.7)	
Hospital mortality: ICU									
All institutions	33/55 (60.0)	37/45 (82.2)	29/59 (49.2)	46/86 (53.5)	55/97 (56.7)	52/86 (60.5)	38/56 (67.9)	25/44 (56.8)	0.921
Successful institutions	21/36 (58.3)	14/21 (66.7)	18/43 (41.9)	24/51 (47.1)	31/55 (56.4)	28/53 (52.8)	21/36 (58.3)	21/36 (58.3)	0.001
Nonsuccessful institutions	12/19 (63.2)	23/24 (95.8)	11/16 (68.8)	22/35(62.9)	24/42 (57.1)	24/33 (72.7)	17/20 (85.0)	4/8 (50.0)	
Percentage of sepsis									
All institutions	199 (51.8)	159 (55.2)	247 (58.5)	344 (64.8)	348 (61.8)	300 (61.0)	270 (67.8)	228 (63.9)	<0.0001
Successful institutions	113 (51.4)	67 (56.8)	143 (59.1)	166 (69.7)	182 (70.0)	133 (61.6)	151 (72.2)	192 (69.1)	<0.0001
Nonsuccessful institutions	86 (52.4)	92 (54.1)	104 (57.8)	178 (60.8)	166 (54.8)	167 (60.5)	119 (63.0)	36 (45.6)	
Time to sepsis diagnosis, h									
All institutions	5.0 (1.5–17.0)	5.3 (1.5–19.5)	3.0 (0.9–10.9)	2.1 (0.9–7.7)	2.2 (0.8–8.7)	2.0 (0.7–6.8)	1.3 (0.6–4.0)	1.0 (0.4–2.1)	<0.0001
Successful institutions	5.7 (1.7–14.8)	5.2 (1.6–14.8)	2.6 (0.9–8.0)	1.7 (0.8–5.3)	2.0 (0.8–5.7)	1.1 (0.5–3.0)	1.0 (0.5–2.6)	0.8 (0.4–2.0)	<0.0001
Nonsuccessful institutions	4.0 (1.3–18.0)	6.3 (1.5–22.0)	4.7 (1.0–13.8)	3.5 (1.0–12.5)	2.9 (0.8–13.5)	3.7 (1.0–14.1)	2.5 (1.0–8.6)	2.0 (1.0–7.5)	
APACHE II score, points									
All institutions	23 (18–30)	23 (18–29)	20 (15–27)	19 (15–24)	18 (14–22)	18 (14–23)	17 (13–22)	17 (13–21)	<0.0001
Successful institutions	21 (17–28)	22 (18–29)	20 (15–25)	18 (14–23)	17 (13–21)	18 (14–22.2)	17 (12–21)	16.5 (13–21)	<0.0001
Nonsuccessful institutions	26 (20–31)	23 (18–28.8)	22 (15.5–28.5)	20 (15–26)	18 (14–22)	19 (14–23)	17 (13–22)	19 (15–23.5)	
SOFA score, points									
All institutions	8 (5–12)	8 (5–11)	7 (5–11)	7 (4–11)	7 (4–11)	7 (4–10)	6 (4–9)	6 (4–10)	<0.0001
Successful institutions	7 (4–11)	8 (5–11)	7 (4–10)	6 (3–9)	6 (3–10)	7 (4–10)	6 (3–9)	6 (3–9)	<0.0001
Nonsuccessful institutions	9 (6–12)	8 (5–11)	8 (5–12)	8 (5–11)	8 (5–12)	7 (5–11)	6 (4–9)	9 (6–12)	
Number of organ dysfunctions									
All institutions	2 (2–3)	2 (2–3)	2 (2–3)	2 (1–3)	2 (1–2)	2 (1–3)	2 (1–2)	2 (1–3)	<0.0001
Successful institutions	2 (2–3)	2 (2–3)	2 (2–3)	2 (1–3)	2 (1–2)	2 (1–2)	2 (1–2)	2 (1–2)	<0.0001
Nonsuccessful institutions	2 (2–3)	2 (2–3)	3 (2–3)	2 (2–3)	2 (1–3)	2 (1–3)	2 (1–3)	2 (2–3)	
Compliance with the first three items^b^									
All institutions	46 (12.0)	37 (12.8)	74 (17.5)	162 (30.5)	183 (32.5)	163 (33.1)	149 (37.4)	151 (42.3)	<0.0001
Successful institutions	21 (9.5)	11 (9.3)	37 (15.3)	70 (29.4)	77 (29.6)	72 (33.3)	70 (33.5)	102 (36.7)	<0.0001
Nonsuccessful institutions	25 (15.2)	26 (15.3)	37 (20.6)	92 (31.4)	106 (35.0)	91 (33.0)	79 (41.8)	49 (62.0)	
Full compliance with 6-h bundle									
All institutions	22 (5.7)	20 (6.9)	41 (9.7)	89 (16.8)	99 (17.6)	82 (16.7)	92 (23.1)	82 (23.0)	<0.0001
Successful institutions	10 (4.5)	8 (6.8)	25 (10.3)	41 (17.2)	37 (14.2)	33 (15.3)	44 (21.1)	59 (21.2)	0.016
Nonsuccessful institutions	12 (7.3)	12 (7.1)	16 (8.9)	48 (16.4)	62 (20.5)	49 (17.8)	48 (25.4)	23 (29.1)	
Compliance with fluids and vasopressors									
All institutions	210 (60.9)	156 (62.2)	271 (76.6)	318 (75.2)	355 (79.1)	323 (81.4)	244 (82.7)	254 (91.0)	<0.0001
Successful institutions	125 (61.0)	55 (51.9)	164 (76.6)	150 (79.4)	164 (82.4)	138 (82.6)	139 (89.7)	203 (95.3)	<0.0001
Nonsuccessful institutions	85 (60.7)	101 (69.7)	107 (76.4)	168 (71.8)	191 (76.4)	185 (80.4)	105 (75.0)	51 (77.3)	
Compliance with antibiotics									
All institutions	218 (56.8)	164 (56.9)	271 (64.2)	358 (67.4)	386 (68.6)	347 (70.5)	274 (68.8)	263 (73.7)	<0.0001
Successful institutions	125 (56.8)	70 (59.3)	167 (69.0)	159 (66.8)	183 (70.4)	158 (73.1)	143 (68.4)	205 (73.7)	0.1072
Nonsuccessful institutions	93 (56.7)	94 (55.3)	104 (57.8)	199 (67.9)	203(67.0)	189 (68.5)	131 (69.3)	58 (73.4)	

*APACHE II* Acute Physiology and Chronic Health Evaluation II, *ED* Emergency department, *ICU* Intensive care unit, *SOFA* Sequential Organ Failure Assessment

^a^General linear models. The first *p* value refers to the comparison throughout the intervention; the second *p* value refers to the comparison between successful and nonsuccessful institutions

^b^Includes lactate sampling, blood cultures, and antibiotics in the first hour

In the logistic regression model, the time to sepsis diagnosis was independently associated with increased mortality (Table 3) in both institution types. By contrast, neither compliance with the entire 6-h bundle nor compliance with antibiotics or fluids and vasopressors (Additional file 1: Table S3) was associated with a reduction in the risk of death. The results of the sensitivity analysis excluding the institutions that did not include patients until the last quarter of the intervention showed similar results (Additional file 1: Table S4). The results of the univariate analysis are available in Additional file 1: Table S5. Other classical variables were also independently associated with a higher risk of death, such as age, cancer and illness severity, and presenting with sepsis in the wards.

**Table 3:** Factors associated with mortality in the whole population and according to the success of the institutions by multivariate analysis

Variables	All institutions		Successful institutions		Nonsuccessful institutions	
	*p* Value	OR (95% CI)	*p* Value	OR (95% CI)	*p* Value	OR (95% CI)
Age, years	<0.0001	1.013(1.008–1.018)	–	–	–	–
SOFA score, points	–	–	0.091	1.038 (0.994–1.083)	<0.0001	1.101 (1.052–1.151)
APACHE II score, points	<0.0001	1.067 (1.049–1.085)	–	–	–	–
Cancer	0.008	1.448 (1.101–1.909)	–	–	–	–
Alcohol abuse			0.001	2.447 (1.442–4.204)	0.931	0.981 (0.637–1.510)
Pneumonia	<0.0001	1.586(1.298–1.940)	–	–	–	–
Abdominal source	–	–	0.750	1.044 (0.799–1.363)	0.005	1.755 (1.198–2.571)
ICU admission	–	–	0.750	1.044 (0.799–1.363)	0.005	0.652 (0.482–0.882)
Septic shock	0.0002	1.634 (1.267–2.108)	–	–	–	–
Sepsis in the wards	0.0034	1.340 (1.102–1.630)	–	–	–	–
Number of organ dysfunctions	<0.0001	1.250 (1.127–1.387)	–	–	–	–
Time to sepsis diagnosis, h	0.0005	1.015 (1.007–1.024)	–	–	–	–
Compliance with the 6-h bundle	0.253	0.864 (0.671–1.109)	–	–	–	–

*SOFA* Sequential Organ Failure Assessment, *APACHE II* Acute Physiology and Chronic Health Evaluation II, *ICU* Intensive care unit

In the multivariate regression model, for the variables that presented a different OR between the successful and nonsuccessful sites, we present the results individually according to the type of site

**Fig. 1:**
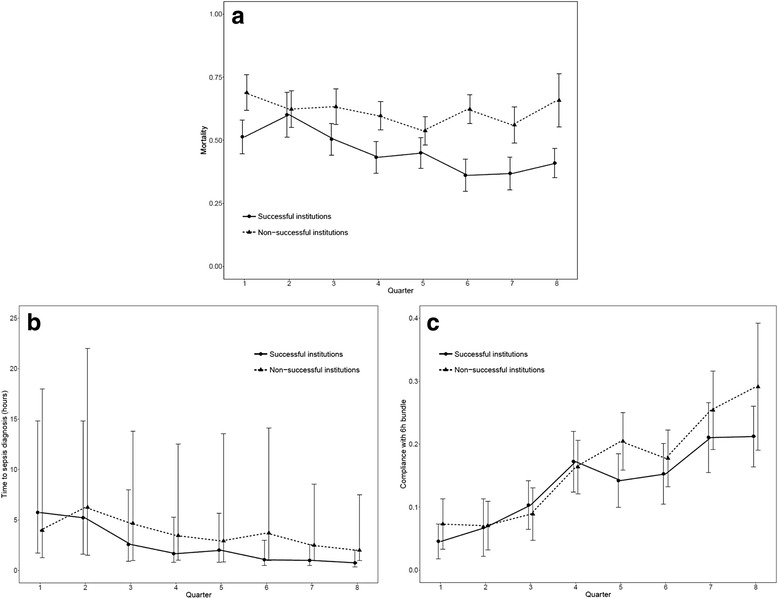
Changes in outcomes throughout the intervention. **a** Hospital mortality. **b** Time to sepsis diagnosis. **c** Full compliance with the 6-h bundle. Generalized linear models for comparison between the two types of institutions: mortality (*p* < 0.0001), time to sepsis diagnosis (*p* < 0.0001), and full compliance with the 6-h bundle (*p* = 0.0160)

Only two institutions had a significant reduction in mortality rates along the quarters (Additional file 1: Table S6 and Figure S1). The reduction in mortality rates can be seen in Fig. 1a. The baseline characteristics of the patients are available in Additional file 1: Table S7. There were some differences in the patient profiles. The percentage of patients from the ED was higher in the successful groups, but these patients also had a higher frequency of comorbidities. These sites were analyzed together (successful institutions) and compared with the institutions without success (nonsuccessful institutions), as shown in Table 2. Although the time to sepsis diagnosis was significantly reduced in both groups, the reduction was greater at the successful sites (Fig. 1b). Although the time curves decayed in parallel, the absolute numbers in the last quarter were smaller in the successful ones. The median time to sepsis diagnosis was still 2.0 (1.0–7.5) in the nonsuccessful hospitals, which was significantly different from the successful ones (0.8 [0.4–2.0], *p* < 0.0001). We also calculated the mean ratio for one-trimester difference for both types of institutions. In the successful ones, the ratio of decline was 0.777 (0.752–0.804, *p* < 0.0001), indicating that there was, on average, a 23% of decline in the time to sepsis diagnosis in each quarter of the intervention. By contrast, the ratio of decline in the nonsuccessful hospitals was 0.868 (0.675–1.116, *p* = 0.270), indicating that the mean ratio of decline did not differ between the quarters. We also found a greater increase in the proportion of patients with sepsis than in those with septic shock at the successful institutions. By contrast, compared with the successful sites, the nonsuccessful sites had a significant increase in compliance with the bundles for both the required items and the entire 6-h bundle (Fig. 1c).

## Discussion

Our results show that the implementation of a sepsis protocol in public institutions in an emerging country led to an overall reduction in the risk of death, proportion of septic shock cases, time to sepsis diagnosis, and improvement in compliance with all 6-h bundle items. These results might be associated with earlier sepsis recognition rather than compliance with treatment indicators. Our findings also suggest that the impact of the implementation of sepsis protocols in public institutions in an emerging country is variable and that mortality reduction is achieved only in some institutions. The successful institutions seemed to be able to reduce the time to sepsis diagnosis more aggressively than the nonsuccessful ones.

Quality improvement initiatives in emerging countries face many challenges [27]. We observed that time to sepsis diagnosis was independently associated with a reduction in the risk of death, although compliance with the 6-h bundle with antibiotics or fluids was not. Although previous studies have demonstrated that compliance with the 6-h bundle and antibiotics was associated with a mortality reduction [15, 28], we were not able to replicate these findings. One explanation is that compliance in this study was measured with time zero beginning at the time of sepsis diagnosis, as acknowledged in the patients’ charts, and not at the time of organ dysfunction onset. Any potential effect of compliance on mortality would be better assessed if compliance were measured according to organ dysfunction onset and not in relation to the moment of sepsis diagnosis. Therefore, compliance alone, not considering the time to sepsis diagnosis, will hardly be associated with mortality reduction in settings where late recognition is frequent. Unfortunately, time zero for calculating compliance is not consistently reported in quality improvement studies. Our findings reinforce the key role of raising awareness, because delayed, although adequate, treatment might not result in optimal survival rates. Another potential explanation is that among the bundle items, some are direct interventions such as antibiotics and others are diagnostic tools, and their impact on mortality will be highly dependent on physicians’ management and not directly on compliance, such as lactate sampling or blood cultures, and we did not assess the adequacy of treatment in a broader way.

As previously shown [29], we observed a trend toward a reduction in disease severity throughout the study period, although none of the hospitals changed their admission profiles. The reduction in the time to sepsis diagnosis could have contributed to a higher percentage of patients being diagnosed with sepsis instead of in the latter stages of septic shock. The early diagnosis also possibly contributed to earlier treatment and therefore could have reduced the odds of progression to septic shock. This hypothesis is reinforced by the finding of a significant reduction in the SOFA score and number of organs with dysfunction. We might argue that a modification in the institution profile could have contributed to these findings. However, there was no relevant change in the baseline characteristics of the patients, except by discreet and irrelevant oscillations in the frequency of some comorbidities. Some differences might have been influenced by our intervention. For example, the increased inclusion of ED patients may indicate a higher awareness of the ED staff. The reduction in the frequency of pneumonia as the source of infection may be a consequence of training and, consequently, the correct diagnosis of respiratory dysfunction secondary to other sources.

The old sepsis definition was used in this study because it was a quality improvement initiative carried out before the new Sepsis 3.0 guidelines definition was available. However, because the Sepsis 3 guidelines define sepsis as the presence of a life-threatening organ dysfunction caused by infection, the definition we used is compatible with this broad concept. The variation in SOFA score, which is the current clinical criterion to define organ dysfunction, is not useful in quality improvement initiatives, because it would require calculations and also, in some cases, time to meet the criteria, which might result in delay in care. As recognized by the Sepsis 3 task force, this was not the intention of the new definition [30]. Thus, the SSC guidelines, on which this quality improvement initiative is based, did not change their criteria even after the launching of the definitions.

Although data from resource-limited settings are scarce, mortality reduction is not a consistent or sustained finding [15, 18, 31]. Our findings support this heterogeneity because only two institutions reduced their mortality rates despite the consistent global improvement in other quality indicators. In the successful institutions, there was a greater reduction in the time to sepsis diagnosis, reinforcing the results of our multivariate analysis. Unfortunately, our study did not address other factors that might influence the feasibility of quality improvement initiatives and help to explain this finding. Difficulties in implementing sepsis protocols have already been reported and include a lack of dedicated staff in the protocol; low availability of resources [32]; shortage of medical and nursing staff [21]; and low compliance with basic principles of quality care, such as continuous training strategies, which is associated with a high professional turnover rate [22], contributing to an inadequate safety culture and low quality of care [33]. These institutional characteristics were not addressed in our study. Also, we did not assess other institutional factors that might be associated with higher sepsis mortality rates, such as the availability of ICU beds or the percentage of patients transferred from other facilities.

Our study has some strengths. First, we reported data from a network of public institutions with a central administration, which is an original approach for a quality improvement initiative in an emerging country. Our results can be generalized to a significant proportion of the Brazilian public healthcare system and potentially to other emerging countries. Second, our implementation strategy was well planned with use of previously validated quality indicators, and we assessed patients in all hospital sectors instead of restricting inclusion to ICU patients. Third, the length of the intervention was sufficiently long to assess the persistence of the effects. However, our study also has some limitations. First, although the LASI provided all tools and information to allow for a similar intervention in all sites, local implementation was highly dependent on the local conditions. We did not measure the percentage of training staff in each site or the number of individually organized meetings. As in most quality improvement initiatives, this step is difficult to measure. Although the audit and feedback provided by the LASI was continuous throughout the intervention, differences in training capacities might have influenced the success of the institutions. The screening process could have been slightly different in some of the hospitals, such that patients might have been missed. Second, we did not monitor the quality of data collection with on-site verification of source documents, although we implemented central monitoring of data for completeness and consistency. Third, the before-after study design precludes any assessment of causality because there was no randomization process or control group. Fourth, we did not measure other concurrent quality improvement processes that might have influenced the results in the successful institutions. In addition, as previously described, we did not assess other information that could explain the differences between institutions, such as the availability of resources, staffing, ICU availability, and other general institutional quality indicators. Organizational factors in each of the institutions, such as nurse/patient ratio, physician and resident staffing hours, the presence of multidisciplinary rounds, the use of protocols in the ICU, or the use of checklists, are not described and might have influenced the results. Fifty, one of the successful sites had baseline mortality rates lower than the other institutions. However, the impact of having a lower baseline mortality rate in a quality improvement initiative is not clear, because this does not necessarily imply that there is a better chance of reducing mortality. We can argue that hospitals with lower mortality are better organized and thus are better settings for quality improvement initiatives. By contrast, this might also mean that the basic adjustments in quality improvement have already been done and that further improvement would be harder to achieve. Along these lines, settings with higher baseline mortality rates might succeed in adjusting basic processes, which will lower their mortality rates.

## Conclusions

A multifaceted approach for sepsis treatment increased compliance and awareness in public institutions in Brazil. However, the impact on mortality was variable. Early recognition seems to be a more relevant factor than compliance with the 6-h bundle in terms of improving the chances of survival.

## Additional file

Additional file 1: Table S1. Definition of compliance with each 6-h bundle item. **Table S2.** Mortality rates throughout the intervention in each of the participating sites according to the location at presentation. **Table S3.** Factors associated with mortality in the whole population, including compliance with antibiotics and fluids/vasopressors. **Table S4.** Factors associated with mortality in the whole population and according to the success of the institutions: multivariate analysis sensitivity analysis without sites 8 and 9. **Table S5.** Global characteristics of the population and risk factors associated with hospital mortality: univariate analysis. **Table S6.** Mortality assessment per site considering the interaction with quarter in the intervention: logistic regression model. **Table S7.** Baseline characteristics of the patients according to the type of institution. **Figure S1.** Hospital mortality rates per quarter of intervention. **a** All sites. **b** Only sites 1 and 5. (DOCX 133 kb)

## Abbreviations

APACHE II: Acute Physiology and Chronic Health Evaluation II
COPD: Chronic obstructive pulmonary disease
CRF: Chronic renal failure
ED: Emergency department
ICU: Intensive care unit
LMICs: Low- and middle-income countries
LASI: Latin American Sepsis Institute
SOFA: Sequential Organ Failure Assessment
SSC: Surviving Sepsis Campaign
