# Supplementary material for: Implementation of sepsis bundles in public hospitals in Brazil: a prospective study with heterogeneous results
Source: Crit Care. 2017 Oct 31;21:268. doi: 10.1186/s13054-017-1858-z (PMC5664817; doi:10.1186/s13054-017-1858-z)
Supplement: Additional file 1: Table S1. — Definition of compliance with each 6-h bundle item. Table S2. Mortality rates throughout the intervention in each of the participating sites according to the location at presentation. Table S3. Factors associated with mortality in the whole population, including compliance with antibiotics and fluids/vasopressors. Table S4. Factors associated with mortality in the whole population and according to the success of the institutions: multivariate analysis sensitivity analysis without sites 8 and 9. Table S5. Global characteristics of the population and risk factors associated with hospital mortality: univariate analysis. Table S6. Mortality assessment per site considering the interaction with quarter in the intervention: logistic regression model. Table S7. Baseline characteristics of the patients according to the type of institution. Figure S1. Hospital mortality rates per quarter of intervention. a All sites. b Only sites 1 and 5. (DOCX 133 kb) [file 13054_2017_1858_MOESM1_ESM.docx]

**Additional file 1**

**Implementation of sepsis bundles in public hospitals in Brazil: a prospective study with heterogeneous results**

Flavia Ribeiro Machado, Elaine Maria Ferreira, Pierre Schippers, Ilusca Cardoso de Paula, Letícia Sandre Vendrame Saes, Francisco Ivanildo de Oliveira Junior, Paula Tuma, Wilson Nogueira Filho, Felipe Piza, Sandra Guare, Cláudia Mangini, Gustavo Ziggiatti Guth, Luciano Cesar Pontes Azevedo, Flavio Geraldo Resende Freitas, Jose Luiz Gomes do Amaral, Nacime Salomão Mansur, Reinaldo Salomão on behalf of the SPDM against sepsis project.

**Definitions**

**Inclusion criteria -** We allowed the institution to include a patient in the database if there was a known or suspected source of infection, associated with at least one sign of systemic inflammatory response syndrome and at least one sepsis-induced organ dysfunction([1](#_ENREF_1)).

**Sepsis-induced organ dysfunction -** We considered organ dysfunction one of the following conditions: hypotension with a systolic blood pressure (SBP) of < 90 mmHg or a mean arterial pressure (MAP) < 65 mmHg or reduction in the preexisting values > 40 mmHg; acute lung injury with hypoxemia, defined as a partial pressure of arterial oxygen/fraction of inspired oxygen (PaO_2_/FiO_2_) ratio of ≤ 300 or need for oxygen supplementation in the presence of pulmonary infiltrates on the x-ray; bilirubin concentrations above 2 mg/dL; low urine output defined as less than 0.5 ml/kg/h for at least one hour or an increase in creatinine above 2.0 mg/dL; coagulopathy, defined by a platelet count < 100,000/mm^3^ or a 50% reduction in the highest number of platelets recorded during the previous three days or a INR > 1.5 or APTT > 60 sec or lactic acidosis with lactate values greater than 1.5-fold the normal value.

**Total number of organ dysfunctions –** number of dysfunctions present on the day of sepsis diagnosis.

**Time to diagnose sepsis -** This time frame was defined as the number of hours between the onset of the first dysfunction and its recognition by the healthcare provider. We defined sepsis recognition as the registration of a sepsis hypothesis in the patient’s chart. To identify the moment of organ dysfunction, the first recording of hypotension, hypoxemia or low urine output as well as the time of the first laboratory test that fulfilled the respiratory, metabolic, coagulation or hepatic criteria for organ dysfunction was retrieved. For patients admitted from the emergency department who already met the criteria for sepsis, we used the time of triage.

**Table S1. Definition of compliance with each 6-hour bundle item**

| **Indicator** | **Description** |
| --- | --- |
| Lactate | Lactate collected within the first 6 hours after organ dysfunction was diagnosed. |
| Blood culture | Blood culture collected before the onset of antibiotic therapy. |
| Antibiotics | Antibiotics administered within one hour of sepsis diagnosis in wards and intensive care units patients and within 3 hours for emergency department patients. |
| Compliance with the obligatory 6-hour bundle | Compliance to lactate sampling, blood cultures and administration of antibiotics. |
| Fluids/vasopressors | Infusion of 20 ml/kg of crystalloids within the first six hours from the diagnosis of sepsis in patients with lactate levels over 2 times the reference level or mean arterial pressure below 65 mmHg AND the use of vasopressor in patients who remain hypotensive after fluid resuscitation. |
| Central venous pressure | Monitoring and maintenance of central venous pressure between 8 – 12 mmHg in patients with spontaneous ventilation or 12 – 15 mmHg in patients under mechanical ventilation who had lactate levels over 2 times the reference level or who required vasopressors to maintain mean arterial pressure above 65 mmHg. |
| Central venous oxygen saturation | Monitoring and maintenance of central venous oxygen saturation above 70% in patients who had lactate levels over 2 times the reference level or who required vasopressors to maintain mean arterial pressure above 65 mmHg. |
| Full 6-hour bundle | Compliance to all items. |

**Table S2 – Mortality rates throughout the intervention in each of the participating sites according to the location at presentation**

| **Variables** | **Baseline**  **n = 384** | **2^nd^ quarter**  **n = 288** | **3^rd^ quarter**  **n = 422** | **4^th^ quarter**  **n = 531** | **5^th^ quarter**  **n = 563** | **6^th^ quarter**  **n = 492** | **7^th^ quarter**  **n = 398** | **8^th^ quarter**  **n = 357** | **Total** |
| --- | --- | --- | --- | --- | --- | --- | --- | --- | --- |
| Site 1 |  |  |  |  |  |  |  |  |  |
| ED (n = 817) | 53/121 (43.8) | 10/28 (35.7) | 41/92 (44.6) | 28/91 (30.8) | 34/104 (32.7) | 27/91 (29.7) | 26/93 (28.0) | 79/197 (40.1) | 298/817 (36.5) |
| Ward (n = 305) | 24/42 (57.1) | 9/18 (50.0) | 36/64 (56.3) | 28/49 (57.1) | 21/39 (53.8) | 10/23 (43.5) | 17/42 (40.5) | 11/28 (39.3) | 156/305 (51.1) |
| ICU (n = 274) | 20/34 (58.8) | 12/18 (66.7) | 17/38 (44.7) | 18/35 (51.4) | 23/44 (52.3) | 21/42 (50.0) | 19/30 (63.3) | 20/33 (60.6) | 150/274 (54.7) |
| All (n = 1396) | 97/197 (49.2) | 31/64 (48.4) | 94/194 (48.5) | 74/175 (42.3) | 78/187 (41.7) | 58/156 (37.2) | 62/165 (37.6) | 110/258 (42.6) | 604/1396 (43.3) |
| Site 2 |  |  |  |  |  |  |  |  |  |
| ED (n = 240) | 11/15 (73.3) | 12/18 (66.7) | 18/33 (54.5) | 28/43 (65.1) | 19/36 (52.8) | 17/27 (63.0) | 30/49 (61.2) | 14/19 (73.7) | 149/240 (62.1) |
| Ward (n = 163) | 7/7 (100) | 8/16 (50.0) | 15/17 (88.2) | 19/31 (61.3) | 16/23 (69.6) | 22/28 (78.6) | 17/32 (53.1) | 4/9 (44.4) | 108/163 (66.3) |
| ICU (n = 26) | 4/4 (100) | 1/1 (100) | 2/3 (66.7) | 5/6 (83.3) | 1/3 (33.3) | 2/2 (100) | 3/4 (75.0) | 2/3 (66.7) | 20/26 (76.9) |
| All (n = 429) | 22/26 (84.6) | 21/35 (60.0) | 35/53 (66.0) | 52/80 (65.0) | 36/62 (58.1) | 41/57 (71.9) | 50/85 (58.8) | 20/31 (64.5) | 277/429 (64.6) |
| Site 3 |  |  |  |  |  |  |  |  |  |
| ED (n = 56) | 6/6 (100) | 6/9 (66.7) | ½(50.0) | 3/6 (50.0) | 1/4 (25.0) | 5/15 (33.3) | 2/10 (20.0) | 2/4 (50.0) | 26/56 (46.4) |
| Ward (n = 74) | 10/12 (83.3) | 8/16 (50.0) | 4/11 (36.4) | 4/4 (100) | 8/16 (50.0) | 5/7 (71.4) | 2/6 (33.3) | 1/2 (50.0) | 42/74 (56.8) |
| ICU (n = 429) | 2/2 (100) | 1/1 (100) | 2/5 (40.0) | 2/6 (33.3) | 3/8 (37.5) | 8/9 (88.9) | 6/8 (75.0) | 2/5 (40.0) | 26/44 (59.1) |
| All (n = 174) | 18/20 (90.0) | 15/26 (57.7) | 7/18 (38.9) | 9/16 (56.3) | 12/28 (42.9) | 18/31 (58.1) | 10/24 (41.7) | 5/11 (45.5) | 94/174 (54.0) |
| Site 4 |  |  |  |  |  |  |  |  |  |
| ED (n = 96) | 3/5 (60.0) | 6/9(66.7) | 10/15 (66.7) | 10/12 (83.3) | 2/10 (20.0) | 21/29 (72.4) | 2/3 (66.7) | 12/13 (92.3) | 66/96 (68.8) |
| Ward (n = 193) | 30/44 (68.2) | 18/32 (56.3) | 26/33 (78.8) | 19/24 (79.2) | 10/27 (37.0) | 14/24 (58.3) | 5/7 (71.4) | 1/2 (50.0) | 123/193 (63.7) |
| ICU (n = 57) | 3/10 (30.0) | 13/13 (100) | 6/6 (100) | 6/11 (54.5) | 7/10 (70.0) | 3/6 (50.0) | 1/1 (100) | 0 | 39/57 (68.4) |
| All (n = 346) | 36/59 61.0) | 37/54 (68.5) | 42/54 (77.8) | 35/47 (74.5) | 19/47 (40.4) | 38/59 (64.4) | 8/11 (72.7) | 13/15 (86.7) | 228/346 (65.9) |
| Site 5 |  |  |  |  |  |  |  |  |  |
| ED (n = 166) | 10/15 (66.7) | 21/27 (77.8) | 9/16 (56.3) | 10/19 (52.6) | 19/34 (55.9) | 8/25 (32.0) | 8/20 (40.0) | 3/10 (30.0) | 88/166 (53.0) |
| Ward (n = 162) | 5/6 (83.3) | 17/24 (70.8) | 18/27 (66.7) | 13/28 (46.4) | 12/28 (42.9) | 5/24 (20.8) | 5/18 (27.8) | 0/7 (0) | 75/162 (46.3) |
| ICU (n = 57) | ½ (50.0) | 2/3 (66.7) | 1/5 (20.0) | 6/16 (37.5) | 8/11 (72.7) | 7/11 (63.6) | 2/6 (33.3) | 1/3 (333.) | 28/57 (49.1) |
| All (n = 385) | 16/23 (69.6) | 40/54 (74.1) | 28/48 (58.3) | 29/63 (46.0) | 39/73 (53.4) | 20/60 (33.3) | 15/44 (34.1) | 4/20 (20.0) | 191/385 (49.6) |
| Site 6 |  |  |  |  |  |  |  |  |  |
| ED (n = 33) | 4/7 (57.1) | 4/7 (57.1) | ½ (50.0) | 3/9 (33.3) | 5/7 (71.4) | 1/1 (100) | - | - | 18/33 (54.5) |
| Ward (n = 55) | 9/13 (69.2) | 5/14 (35.7) | 4/7 (57.1) | 4/8 (50.0) | 5/8 (62.5) | 5/5 (100) | - | - | 32/55 (58.2) |
| ICU (n = 22) | 1/1 (100) | 4/5 (80.0) | 1/1 (100) | 3/4 (75.0) | 5/8 (62.5) | 3/3 (100) | - | - | 17/22 (77.3) |
| All (n = 110) | 14/21 (66.7) | 13/26 (50.0) | 6/10 (60.0) | 10/21 (47.6) | 15/23 (65.2) | 9/9 (100) | - | - | 67/110 (60.9) |
| Site 7 |  |  |  |  |  |  |  |  |  |
| ED (n = 67) | 3/8 (37.5) | 2/2 (100) | 2/4 (50.0) | 4/9 (44.4) | 17/23 (73.9) | 8/15 (53.3) | 3/6 (50.0) | - | 39/67 (58.2) |
| Ward (n = 31) | 7/9 (77.8) | 1/1 (100) | 1/2 (50.0) | 4/6 (66.7) | 4/7 (57.1) | 2/4 (50.0) | 2/2 (100) | - | 21/31 (67.7) |
| ICU (n = 9) | 0 | 3/3 (100) | 1/1 (100) | 3/5 (60.0) | 0 | 0 | 0 | - | 7/9 (77.8) |
| All (n = 107) | 10/17 (58.8) | 6/6 (100) | 3/6 (50.0) | 9/16 (56.3) | 24/35 (68.6) | 10/19 (52.6) | 5/8 (62.5) | - | 67/107 (62.6) |
| Site 8 |  |  |  |  |  |  |  |  |  |
| ED (n = 88) | 1/2 (50.0) | 0 | 3/8 (37.5) | 9/18 (50.0) | 9/24 (37.5) | 5/15 (33.3) | 5/16 (31.3) | 3/5 (60.0) | 35/88 (39.8) |
| Ward (n = 140) | 6/10 (60.0) | 0 | 4/6 (66.7) | 19/40 (47.5) | 20/29 (69.0) | 19/29 (65.5) | 11/14 (78.6) | 8/12 (66.7) | 87/140 (62.1) |
| ICU (n = 31) | 2/2 (100) | 1/1 (100) | 0/1 (0) | 1/2 (50) | 4/6 (66.7) | 8/12 (66.7) | 7/7 (100) | 0 | 23/31 (74.2) |
| All (n = 259) | 9/14 (64.3) | 1/1 (100) | 7/15 (46.7) | 29/60 (48.3) | 33/59 (55.9) | 32/56 (57.1) | 23/37 (62.2) | 11/17 (64.7) | 145/259 (56.0) |
| Site 9 |  |  |  |  |  |  |  |  |  |
| ED (n = 66) | 3/5 (60.0) | 8/14 (57.1) | 6/7 (85.7) | 9/17 (52.9) | 3/5 (60.0) | 5/14 (35.7) | 2/4 (50.0) | - | 36/66 (54.5) |
| Ward (n = 155) | 1/2 (50.0) | 5/8 )62.5) | 8/17 (47.1) | 18/31 (58.1) | 20/42 (47.6) | 19/30 (63.3) | 8/20 (40.0) | 3/5 (60.0) | 82/155 (52.9) |
| ICU (n = 8) | 0 | 0 | 0 | 4/5 (80.0) | 1/2 (50.0) | 0/1 (0) | 0 | 0 | 5/8 (62.5) |
| All (n = 229) | 4/7 (57.1) | 13/22 (59.1) | 14/24 (58.3) | 31/53 (58.5) | 24/49 (49.0) | 24/45 (53.3) | 10/24 (41.7) | 3/5 (60.0) | 123/229 (53.7) |
| Successful institutions |  |  |  |  |  |  |  |  |  |
| ED (n = 983) | 63/136 (46.3) | 31/55 (56.4) | 50/108 (46.3) | 38/110 (34.5) | 53/138 (38.4) | 35/116 (30.2) | 34/113 (30.1) | 82/207 (39.6) | 386/983 (39.3) |
| Ward (n = 467) | 29/48 (60.4) | 26/42 (61.9) | 54/91 (59.3) | 41/77 (53.2) | 33/67 (49.3) | 15/47 (31.9) | 22/60 (36.7) | 11/35 (31.4) | 231/467 (49.5) |
| ICU (n = 331) | 21/36 (58.3) | 14/21 (66.7) | 18/43 (41.9) | 24/51 (47.1) | 31/55 (56.4) | 28/53 (52.8) | 21/36 (58.3) | 21/36 (58.3) | 178/331 (53.8) |
| All (n = 1781) | 113/220 (51.4) | 71/118 (60.2) | 122/242 (50.4) | 103/238 (43.3) | 117/260 (45.0) | 78/216 (36.1) | 77/209 (36.8) | 114/278 (41.0) | 795/1781 (44.6) |
| Unsuccessful institutions |  |  |  |  |  |  |  |  |  |
| ED (n = 646) | 31/48 (64.6) | 38/59 (64.4) | 41/71 (57.7) | 66/114 (57.9) | 56/109 (51.4) | 62/116 (53.4) | 44/88 (50.0) | 31/41 (75.6) | 369/646 (57.1) |
| Ward (n = 811) | 70/97 (72.2) | 45/87 (51.7) | 62/93 (66.7) | 87/144 (60.4) | 83/152 (54.6) | 86/127 (67.7) | 45/81 (55.6) | 17/30 (56.7) | 495/811 (61.0) |
| ICU (n = 197) | 12/19 (63.2) | 23/24 (95.8) | 11/16 (68.8) | 22/35(62.9) | 24/42 (57.1) | 24/33 (72.7) | 17/20 (85.0) | 4/8 (50.0) | 137/197 (69.5) |
| All (n = 1654) | 113/165 (68.9) | 106/170 (62.4) | 114/180 (63.3) | 175/293(59.7) | 163/303 (53.8) | 172/276 (62.3) | 106/189 (56.1) | 52/79 (65.8) | 1001/1654 (60.5) |
| All institutions |  |  |  |  |  |  |  |  |  |
| ED (n = 1629) | 94/184 (51.1) | 69/114 (60.5) | 91/179 (50.8) | 104/224 (46.4) | 109/247 (44.1) | 97/232 (41.8) | 78/201 (38.8) | 113/248 (45.6) | 755/1629 (46.3) |
| Ward (n = 1228) | 99/145 (68.3) | 71/129 (55.0) | 116/184 (63.0) | 128/221 (57.9) | 116/219 (53.0) | 101/174 (58.0) | 67/141 (47.5) | 28/65 (43.1) | 726/1278 (56.8) |
| ICU (n = 528) | 33/55 (60.0) | 37/45 (82.2) | 29/59 (49.2) | 46/86 (53.5) | 55/97 (56.7) | 52/86 (60.5) | 38/56 (67.9) | 25/44 (56.8) | 315/528 (59.7) |

APACHE - *Acute Physiologic Chronic Health Evaluation*; SOFA - *Sequential Organ Failure Assessment.* ^a^ General linear models. The first p value refers to the comparison throughout the intervention, the second p value refers to the comparison between successful and non-successful institutions. ^b^ includes lactate sampling. blood cultures and antibiotics in the first hour.

**Table S3 – Factors associated with mortality in the whole population including compliance with antibiotics and fluids/vasopressors.**

| **Variables** | **All institutions** | | **Successful institutions** | | **Non-successful institutions** | |
| --- | --- | --- | --- | --- | --- | --- |
|  | **P value** | **OR (CI95%)** | **P value** | **OR (CI95%)** | **P value** | **OR (CI95%)** |
| Age (years) | < 0.0001 | 1.017(1.012 - 1.022) | - | - | - | - |
| SOFA (points) | < 0.0001 | 1.074 (1.040 - 1.109) | - | - | - | - |
| APACHE II (points) | < 0.0001 | 1.053 (1.036 - 1.071) | - | - | - | - |
| Alcoholism | 0.0199 | 1.529 (1.073 – 2.196) | - | - | - | - |
| Pneumonia | 0.0001 | 1.504 (1.230 – 1.839) | - | - | - | - |
| Abdominal source | - | - | 0.804 | 0.955 (0.664 - 1.373) | 0.001 | 2.047(1.332 – 3.148) |
| Septic shock | 0.0001 | 1.619 (1.280 – 2.048) | - | - | - | - |
| Sepsis in the wards | 0.0066 | 1.322 (1.081 – 1.618) | - | - | - | - |
| Number of organ dysfunctions | 0.0001 | 1.228 (1.107 - 1.363) | - | - | - | - |
| Time to sepsis diagnosis (h) | 0.0008 | 1.014 (1.006 - 1.022) | - | - | - | - |
| Compliance with the 6h bundle | 0.0811 | 0.750 (0.541 - 1.034) | - | - | - | - |

OR - *odds ratio*; CI95% - confidence interval; SOFA - *Sequential Organ Failure Assessment;* APACHE II – *Acute Physiology and Chronic Health Evaluation II*. Multivariate regression model: for the variables that presented a different odds ratio between the successful and non-successful sites, we presented the results individually according to the type of sites.

**Table S4 – Factors associated with mortality in the whole population and according to the success of the institutions – multivariate analysis sensitivity analysis without sites 8 and 9**

| **Variables** | **All institutions** | | **Successful institutions** | | **Non-successful institutions** | |
| --- | --- | --- | --- | --- | --- | --- |
|  | **P value** | **OR (CI95%)** | **P value** | **OR (CI95%)** | **P value** | **OR (CI95%)** |
| Age (years) | < 0.0001 | 1.014(1.091 - 1.904) | - | - | - | - |
| SOFA (points) | 0.0008 | 1.062 (1.025 - 1.101) | - | - | - | - |
| APACHE II (points) | < 0.0001 | 1.068 (1.049 - 1.087) | - | - | - | - |
| Cancer | 0.0101 | 1.440 (1.091 - 1.904) | - | - | - | - |
| Alcoholism | - | - | 0.0009 | 2.481 (1.454 – 4.281) | 0.7780 | 0.937 (0.595 0 1.475) |
| Pneumonia | < 0.0001 | 1.573 (1.280 – 1.936) | - | - | - | - |
| Abdominal source | - | - | 0.685 | 0.925 (0.632 - 1.348) | 0.0051 | 1.753(1.184 - 2.595) |
| ICU admission | - | - | 0.853 | 0.975 (0.749 - 1.268) | 0.008 | 0.660 (0.486 - 0.897) |
| Septic shock | 0.0013 | 1.537 (1.183 – 1.996) | - | - | - | - |
| Sepsis in the wards | 0.0018 | 1.381 (1.128 - 1.692) | - | - | - | - |
| Number of organ dysfunctions | < 0.0001 | 1.283 (1.153 - 1.429) | - | - | - | - |
| Time to sepsis diagnosis (h) | 0.0002 | 1.017 (1.008 - 1.026) | - | - | - | - |
| Compliance with the 6h bundle | 0.179 | 0.838 (0.646 - 1.084) | - | - | - | - |

OR - *odds ratio*; CI95% - confidence interval; SOFA - *Sequential Organ Failure Assessment;* APACHE II – *Acute Physiology and Chronic Health Evaluation II*; ICU – intensive care unit. Multivariate regression model: for the variables that presented a different odds ratio between the successful and non-successful sites, we presented the results individually according to the type of sites.

**Table S5 – Global characteristics of the population and risk factors associated with hospital mortality – univariate analysis**

| **Variable** | **All patients**  **(n = 3435)** | **Survivors**  **(n = 1639)** | **Non-survivors**  **(n = 1796)** | **P value^a^** |
| --- | --- | --- | --- | --- |
| Type of institution |  |  |  | < 0.001 |
| Successful | 1781/3435 (51.8) | 986/1639 (60.2) | 795/1796 (44.3) |  |
| Non-successful | 1654/3435 (48.2) | 653/1639 (39.8) | 1001/1796 (55.7) |  |
| Age (years) | 61 (47 – 74) | 56 (40 – 71) | 65 (53 – 76) | < 0.001 |
| Male gender | 1933/3435 (56.3) | 927/1639 (56.6) | 1006/1796 (56.0) | 0.748 |
| APACHE II (points) | 19 (14 – 24) | 16 (12 – 21) | 21 (17 – 27) | <0.001 |
| SOFA score (points) | 7 (4 – 11) | 5 (3 – 8) | 9 (6 – 12) | < 0.001 |
| Comorbidities |  |  |  |  |
| COPD | 256/3435 (7.5) | 115/1639 (7.0) | 141/1796 (7.9) | 0.352 |
| Cancer | 414/3435 (12.1) | 174/1639 (10.6) | 240/1796 (13.4) | 0.014 |
| Chronic renal failure | 407/3435 (11.8) | 180/4639 (11.0) | 227/1796 (12.6) | 0.133 |
| Arterial hypertension | 1450/3435 (42.2) | 628/1639 (38.3) | 822/1796 (45.8) | <0.001 |
| Alcoholism | 299/3435 (8.7) | 116/1639 (7.1) | 183/1796 (10.2) | 0.001 |
| Immunosuppression | 638/3435 (18.6) | 291/1639 (17.8) | 347/1796 (19.3) | 0.238 |
| Diabetes mellitus | 842/3435 (24.5) | 377/1639 (23.0) | 465/1796 (25.9) | 0.049 |
| Obesity | 79/3435 (2.3) | 27/1639 (1.6) | 52/1796 (2.9) | 0.015 |
| Type of admission |  |  |  | 0.628 |
| Medical | 2567/3435 (74.7) | 1231/1639 (75.1) | 1336/1796 (74.4) |  |
| Surgical | 868/3432 (25.3) | 408/1639 (24.9) | 460/1796 (25.6) |  |
| ICU admission | 1930/3435 (56.1) | 832/1639 (50.8) | 1098/1796 (61.1) | <0.0001 |
| Severity of illness |  |  |  | <0.0001 |
| Sepsis | 2095/3435 (61.0) | 1246/1639 (76.0) | 849/1796 (47.3) |  |
| Septic shock | 1340/3435 (39.0) | 393/1639 (24.0) | 947/1796 (52.7) |  |
| Type of infection |  |  |  | <0.001 |
| Community | 1998/3435 (58.2) | 1031/1639 (62.9) | 967/1796 (53.8) |  |
| Nosocomial | 1437/3435 (41.8) | 608/1639 (37.1) | 829/1796 (46.2) |  |
| Location at sepsis presentation |  |  |  | <0.001 |
| Emergency department | 1629/3435 (47.4) | 874/1639 (53,3) | 755/1796 (42.0) |  |
| Wards | 1278/3435 (37.2) | 552/1639 (33.7) | 726/1796 (40.4) |  |
| ICU | 528/3435 (15.4) | 213/1639 (13.0) | 315/1796 (17.5) |  |
| Source of infection |  |  |  |  |
| Lung | 1927/3435 (56.1) | 844/1639 (51.5) | 1083/1796 (60.3) | <0.001 |
| Intraabdominal | 534/3435 (15.5) | 241/1639 (14.7) | 293/1796 (16.3) | 0.193 |
| Urinary tract | 572/3435 (16.7) | 309/1639 (18.9) | 263/1796 (14.6) | 0.001 |
| Organ dysfunction (number) |  |  |  |  |
| Cardiovascular | 2480/3435 (72.2) | 1072/1639 (65.4) | 1408/1796 (78.4) | <0.001 |
| Renal | 846/3435 (24.6) | 313/1639 (19.1) | 533/1796 (29.7) | <0.001 |
| Respiratory | 1989/3435 (57.9) | 871/1639 (53.1) | 1118/1796 (62.2) | <0.001 |
| Coagulation | 642/3435 (18.7) | 227/1639 (13.8) | 415/1796 (23.1) | <0.001 |
| Hepatic | 325/3435 (9.5) | 115/1639 (7.0) | 210/1796 (11.7) | <0.001 |
| Lactate ≥ 2 mmol/L | 977/2966 (32.9) | 578/1413 (37.2) | 399/1553 (28.2) | <0.001 |
| Lactate ≥ 4 mmol/L | 539/2966 (18.2) | 170/1413 (12.0) | 369/1553 (23.8) | <0.001 |
| Number of organ dysfunction | 2 (1 – 3) | 2 (1 – 2) | 2 (2 – 3) | <0.001 |
| Time to sepsis diagnosis (h) | 2.1 (0.8 – 8.0) | 1.6 (0.7 – 5.3) | 3.0 (1.0 – 12.3) | <0.001 |
| Diagnosis at weekends/night | 1602/3435 (46.6) | 747/1639 (45.6) | 855/1796 (47.6) | 0.234 |
| Lactate sampling | 2601/3435 (75.7) | 1274/1639 (77.7) | 1327/1796 (73.9) | 0.009 |
| Blood cultures sampling | 1884/3435 (54.8) | 902/1639 (55.0) | 982/1796 (54.7) | 0.834 |
| Early antibiotics | 2281/3435 (66.4) | 1127/1639 (68.8) | 1154/1796 (64.3) | 0.005 |
| Compliance with the first 3 items^b^ | 965/3435 (28.1) | 478/1639 (29.2) | 487/1796 (27.1) | 0.182 |
| Fluíds and vasopressors | 2131/2793 (76.3) | 926/1235 (75.0) | 1205/1558 (77.3) | 0.145 |
| CVP optimization | 121/2117 (5.7) | 40/838 (4.8) | 81/1279 (6.3) | 0.131 |
| ScvO_2_ optimization | 24/2117 (1.1) | 8/838 (1.0) | 16/1279 (1.3) | 0.318 |
| Compliance with 6h bundle | 527/3435 (15.3) | 322/1639 (19.6) | 205/1796 (11.4) | <0.001 |

APACHE - Acute Physiologic Chronic Health Evaluation; SOFA - Sequential Organ Failure Assessment; COPD – chronic obstructive pulmonary disease; ICU – intensive care unit; CPV – central venous pressure; SvcO_2_ – central venous oxygen saturation. Results expressed in number (%) or median (25% - 75%). ^a^Chi-square and Mann Whitney. ^b^ includes lactate sampling, blood cultures and antibiotics in the first hour.

**Table S6. Mortality assessment per site considering the interaction with quarter in the intervention – logistic regression model**

| **Variable** | **P-Value** | **Odds ratio** | **Confidence interval** | |
| --- | --- | --- | --- | --- |
|  |  |  | **(95%)** | |
|  |  |  | **Upper Limit** | **Lower Limit** |
| **Quarter:site1** | **0.013** | **0.945** | **0.903** | **0.988** |
| Quarter:site2 | 0.937 | 0.996 | 0.891 | 1.111 |
| Quarter:site3 | 0.130 | 0.891 | 0.765 | 1.033 |
| Quarter:site4 | 0.338 | 1.060 | 0.941 | 1.195 |
| **Quarter:site5** | **< 0.001** | **0.772** | **0.682** | **0.871** |
| Quarter:site6 | 0.100 | 1.224 | 0.964 | 1.563 |
| Quarter:site7 | 0.896 | 1.015 | 0.81 | 1.263 |
| Quarter:site8 | 0.128 | 1.128 | 0.966 | 1.32 |
| Quarter:site9 | 0.661 | 0.963 | 0.814 | 1.137 |

Site 1 and site 5 were considered as successful institutions. The OR shows a reduction in the risk of dying by each quarter the institutions was in the intervention. All the other sites were considered non-successful institutions as there was not reduction in the risk of death by quarter the institutions remained in the intervention.

**
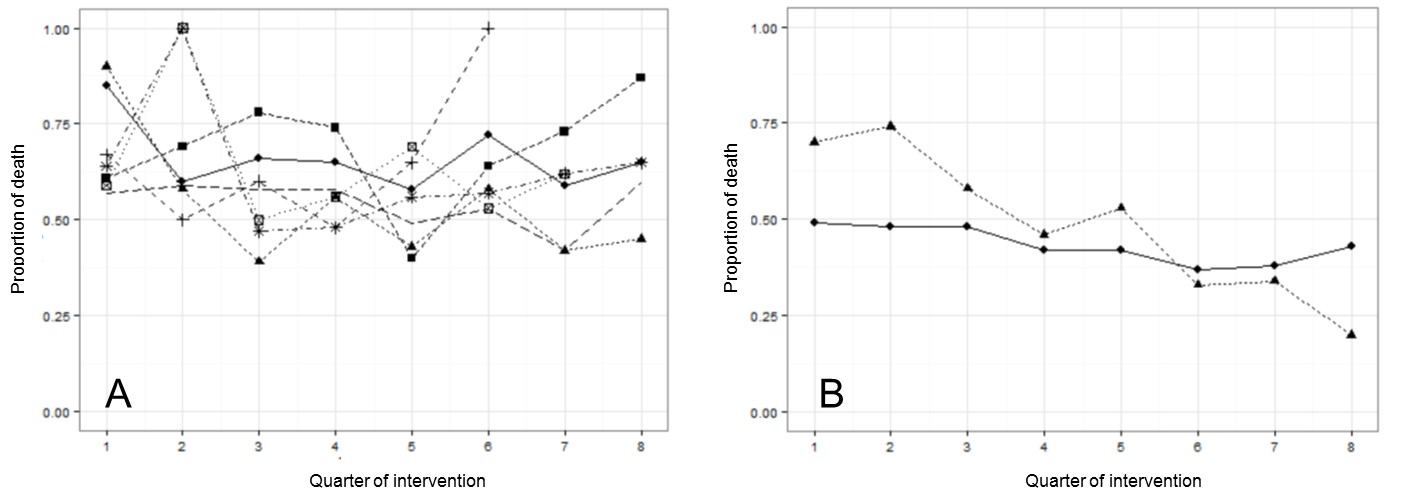
**

**Figure S1. Hospital mortality rates per quarter of intervention.** Panel A – All sites. Panel B. Only sites 1 and 5

**Table S7 – Baseline characteristics of the patients according to the type of institution**

| **Variable** | **Successful institutions**  **(n = 1781)** | **Non-successful institutions**  **(n = 1654)** | **P value^a^** |
| --- | --- | --- | --- |
| Age (years) | 61 (47 – 74) | 61 (46 – 75) | 0.945 |
| Male gender | 971/1781 (54,5) | 962/1654 (58.2) | 0.032 |
| Comorbidities |  |  |  |
| COPD | 165/1781 (9.3) | 91/1654 (5.5) | <0.0001 |
| Cancer | 241/1781 (13.5) | 173/1654 (10.5) | 0.006 |
| Chronic renal failure | 242/1781 (13.6) | 165/1654 (10.0) | 0.001 |
| Arterial hypertension | 738/1781 (41.4) | 712/1654 (43.0) | 0.340 |
| Alcoholism | 90/1781 (5.1) | 209/1654 (12.6) | <0.0001 |
| Immunosuppression | 386/17811 (21.7) | 252/1654 (15.2) | 0.001 |
| Diabetes mellitus | 418/1781 (23.5) | 424/1654 (25.6) | 0.141 |
| Obesity | 28/1781 (1.6) | 51/1654 (3.1) | 0.003 |
| Type of admission |  |  | 0.006 |
| Medical | 1366/1781 (76.7) | 1201/1654 (72.6) |  |
| Surgical | 415/1781 (23.3) | 453/1654 (27.4) |  |
| ICU admission | 990/1781 (55.6) | 940/1654 (56.8) | 0.462 |
| Type of infection |  |  | 0.839 |
| Community | 1033/1781 (58.0) | 965/1654 (58.3) |  |
| Nosocomial | 748/1781 (42.0) | 689/1654 (41.7) |  |
| Location at sepsis presentation |  |  | <0.001 |
| Emergency department | 983/1781 (55.2) | 646/1654 (39.1) |  |
| Wards | 467/1781 (26.2) | 811/1654 (49.0) |  |
| ICU | 331/1781 (18.6) | 197/1654 (11.9) |  |
| Source of infection |  |  |  |
| Lung | 983/1781 (55.2) | 944/1654 (57.1) | 0.267 |
| Intraabdominal | 248/1781 (13.9) | 286/1654 (17.3) | 0.007 |
| Urinary tract | 253/1781 (14.2) | 319/1654 (19.3) | <0.001 |

COPD – chronic obstructive pulmonary disease; ICU – intensive care unit. Results expressed in number (%) or median (25% - 75%). ^a^Chi-square and Mann Whitney.
